# Selenium Augments microRNA Directed Reprogramming of Fibroblasts to Cardiomyocytes via Nanog

**DOI:** 10.1038/srep23017

**Published:** 2016-03-15

**Authors:** Xiaowen Wang, Conrad P Hodgkinson, Kefeng Lu, Alan J Payne, Richard E Pratt, Victor J Dzau

**Affiliations:** 1 Mandel Center for Hypertension Research and Division of Cardiovascular Medicine, Department of Medicine, Duke University Medical Center, Durham, NC 27710, USA; 2 Department of Cardiology, Shandong Provincial Hospital, Jinan, Shandong 250001, China

## Abstract

We have recently shown that a combination of microRNAs, miR combo, can directly reprogram cardiac fibroblasts into functional cardiomyocytes *in vitro* and *in vivo*. However, direct reprogramming strategies are inefficient and slow. Moving towards the eventual goal of clinical application it is necessary to develop new methodologies to overcome these limitations. Here, we report the identification of a specific media composition, reprogramming media (RM), which augmented the effect of miR combo by 5–15-fold depending upon the cardiac marker tested. RM alone was sufficient to strongly induce cardiac gene and protein expression in neonatal tail-tip as well as cardiac fibroblasts. Expression of pluripotency markers Nanog, Oct4, Sox2, and Klf4 was significantly enhanced by RM, with miR combo augmenting the effect further. Knockdown of Nanog by siRNA inhibited the effect of RM on cardiac gene expression. Removal of insulin-transferrin-selenium completely inhibited the effect of reprogramming media upon cardiac gene expression and the addition of selenium to standard culture media recapitulated the effects of RM. Moreover, selenium enhanced the reprogramming efficiency of miR combo.

Following cardiac injury in adult mammals cardiomyocytes are lost but not replaced in significant numbers that suffice to contribute to significant heart regeneration^1^. The cardiac fibroblast population significantly expands, promoting excessive fibrosis and scar formation^2^. Fibrotic remodeling of the injured myocardium negatively impacts contractility and electrical conduction. Over time this leads to further deterioration in heart function and eventual organ failure^2^. Converting cardiac fibroblasts within the scar tissue into cardiomyocytes would be expected to improve heart function. Consequently, identifying ways to generate cardiomyocytes from fibroblasts has become a significant endeavor in regenerative medicine. To date, cardiomyocytes have been generated from fibroblasts by direct and indirect reprogramming approaches. Indirect reprogramming involves an intermediary step, fibroblasts are first converted into iPS cells and these iPS cells are subsequently differentiated into cardiomyocytes. In contrast, direct reprogramming does not require an intermediary step and the fibroblasts are directly converted into cardiomyocytes. Direct reprogramming is the preferred approach for safety and ethical concerns. For example, direct reprogramming removes the necessity to use oncogenic proteins such as Klf4 and c-Myc which are needed to force cells to adopt an iPS state. We, and a number of other researchers, have identified distinct sets of microRNAs and/or transcription factors that directly reprogrammed cardiac fibroblasts into cardiomyocytes^3^,^4^,^5^,^6^,^7^,^8^. Importantly microRNA and transcription factor mediated direct reprogramming of fibroblasts into cardiomyocytes promoted functional recovery in the infarcted heart^3^,^5^,^7^,^8^. However, a significant issue with direct reprogramming is low efficiency which negatively impacts clinical applications of this technique. Identifying strategies that improve efficiency is important for the field to advance towards a clinical application of direct reprogramming.

Here we report the identification of a chemically defined media that increased the efficiency of microRNA directed reprogramming by elevating base-line cardiac gene and protein expression. This reprogramming media promoted cardiac gene and protein expression in both cardiac and tail-tip fibroblasts via Nanog. Selenium was identified as the active ingredient.

## Results

### Enhancing microRNA mediated direct reprogramming of fibroblasts to cardiomyocytes

We have previously shown that a combination of microRNAs, which we called miR combo (miR-1, miR-133, miR-208, miR-499), directly reprograms cardiac fibroblasts into cardiomyocytes both *in vitro* and *in vivo*^3^,^5^. In this study we sought to identify a method that would enhance the efficiency of microRNA directed reprogramming of fibroblasts into cardiomyocyte-like cells *in vitro*^3^,^5^.

Previous work in our laboratory has shown that a media composed of Advanced DMEM/F12, ascorbic acid and insulin-transferrin-selenium promotes the differentiation of Sca-1^+^ cardiac progenitors into cardiomyocyte-like cells^9^. On the basis that cardiac progenitors and fibroblasts have many surface markers in common we hypothesized that this media would augment miR combo direct reprogramming.

**Figure 1:**
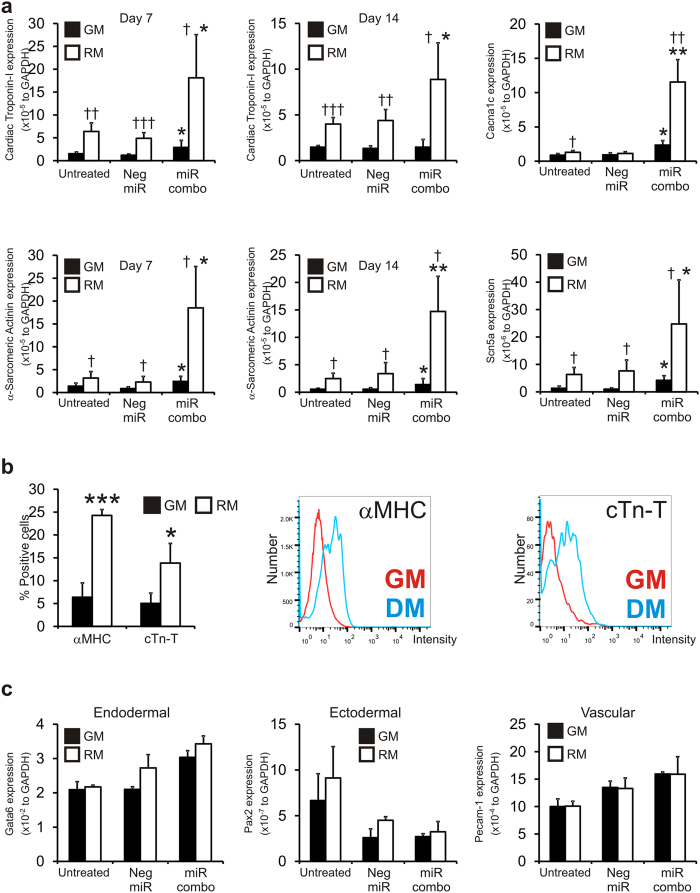
Reprogramming media augments the effect of miR combo in neonatal cardiac fibroblasts. (**a**) Neonatal cardiac fibroblasts were transfected with vehicle, negative control miR (Neg-miR) or miR combo. The day after transfection the cells were cultured in either growth media (GM) or reprogramming media (RM) for the indicated times. Expression of cardiac markers was determined by qPCR and is expressed relative to the endogenous control GAPDH. N = 3–7. *Comparisons made between miR combo and negative control miR ***P < 0.001, **P < 0.01, *P < 0.05. ^†^Comparisons made between reprogramming media and growth media for each group ^†††^P < 0.001, ^††^P < 0.01, ^†^P < 0.05. (**b**) Neonatal cardiac fibroblasts were cultured in either growth media (GM) or reprogramming media (RM) for 14 days. Protein expression of the cardiac specific markers α-myosin heavy chain (αMHC) and cardiac troponin-T (cTn-T) was determined by flow cytometry. N ≥ 6. ***P < 0.001, *P < 0.05. (**c**) Neonatal cardiac fibroblasts were transfected with vehicle, negative control miR (Neg-miR) or miR combo. The day after transfection the cells were cultured in either growth media (GM) or reprogramming media (RM) for the indicated times. Expression of endodermal, ectodermal and vascular markers was determined by qPCR and is expressed relative to the endogenous control GAPDH. N = 3. *Comparisons made between miR combo and negative control miR ***P < 0.001, **P < 0.01, *P < 0.05. ^†^Comparisons made between reprogramming media and growth media for each group ^†††^P < 0.001, ^††^P < 0.01, ^†^P < 0.05.

Neonatal cardiac fibroblasts were transfected with a negative control miR (negmiR) or miR combo. Cells were then cultured for 7 or 14 days in growth media (GM) or reprogramming media (RM). In-line with our previous findings, in standard growth media (GM) miR combo significantly induced the expression of a number of markers that are found in mature cardiomyocytes (Fig. 1a). Reprogramming media (RM) significantly augmented the effect of miR combo for all of the cardiac markers tested with a fold increase of 2- to 20-fold (Fig. 1a). Similarly, in both the mock and negmiR transfected fibroblasts cardiac gene expression was similarly 2- to 10-fold higher in the RM treated group when compared to the GM treated group (Fig. 1a). Thus, RM was augmenting the effect of miR combo by increasing baseline cardiac gene expression. Changes in gene expression are not necessarily reflected in protein levels so we measured Cardiac Troponin-T and α-MHC protein expression by flow cytometry. After 14 days neonatal cardiac fibroblasts cultured in RM were found to have significantly higher levels of Cardiac Troponin-T and α-MHC protein when compared to the growth media treated group (Fig. 1b). Finally, we assessed the specificity of reprogramming media by measuring the expression of ectoderm, endoderm and vascular markers. Reprogramming media did not increase the expression of the endodermal markers Gata6 and Sox17 (Fig. 1c and Supplementary Fig. 1). Similarly the expression of ectodermal (Pax-2, Pax-6) and vascular markers (Pecam-1, Flk-1) were unaffected by reprogramming media (Fig. 1c and Supplementary Fig. 1).

**Figure 2:**
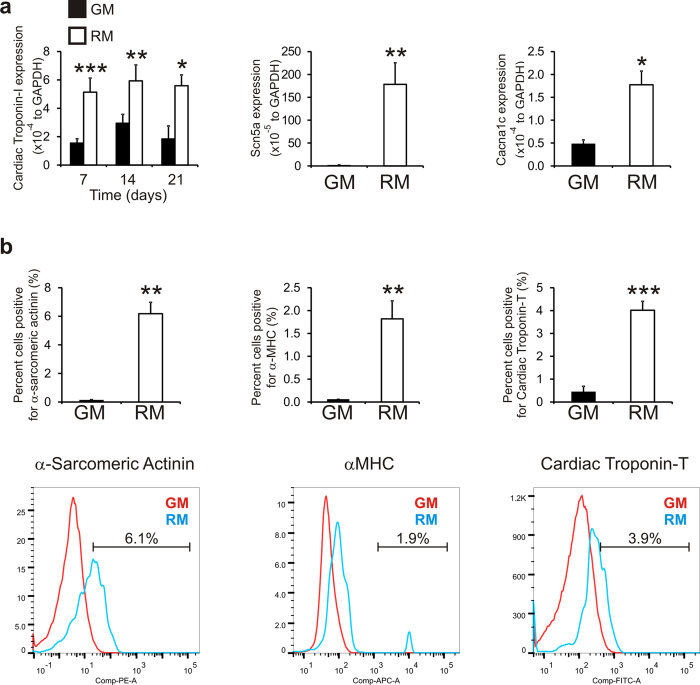
Reprogramming media induces cardiac gene expression in neonatal tail-tip fibroblasts. (**a**) Neonatal tail-tip fibroblasts were cultured in either growth media (GM) or reprogramming media (RM) for 14 (Cardiac Troponin-I) or 21 days (Scn5a, Cacna1c). Expression of the cardiac specific markers was determined by qPCR and is expressed relative to the endogenous control GAPDH. N = 3–11. ***P < 0.001, **P < 0.01, *P < 0.05. (**b**) Neonatal tail-tip fibroblasts were cultured in either growth media (GM) or reprogramming media (RM) for 14 days. Protein expression of the cardiac specific markers α-sarcomeric actinin, α-myosin heavy chain and cardiac troponin-T was determined by flow cytometry. N = 3. Representative traces are shown.

To eliminate the possibility that our results were due to the differentiation of cardiac progenitors within our cardiac fibroblast isolations we repeated our experiments with neonatal tail-tip fibroblasts. Neonatal tail-tip fibroblasts were isolated and cultured in either fibroblast growth media (GM) or reprogramming media (RM). We first evaluated gene expression by qPCR. RM significantly increased the mRNA levels of Cardiac Troponin-I, an intermediate cardiomyocyte marker, at all time-points tested (Fig. 2a). Similarly, RM robustly stimulated expression of cardiomyocyte sodium (Scn5a) and calcium (Cacna1c) channels (Fig. 2a). We then determined whether the changes in cardiac gene expression were reflected at the protein level. Neonatal tail-tip fibroblasts were cultured for 14 days in either GM or RM. Cells were stained with antibodies for cardiac markers and then analyzed by flow cytometry. RM increased the number of cells expressing α-sarcomeric actinin, α-myosin heavy chain, and cardiac troponin-T by greater than 5-fold, when compared to the GM treated group (Fig. 2b).

### Nanog is important for the effects of reprogramming media

**Figure 3:**
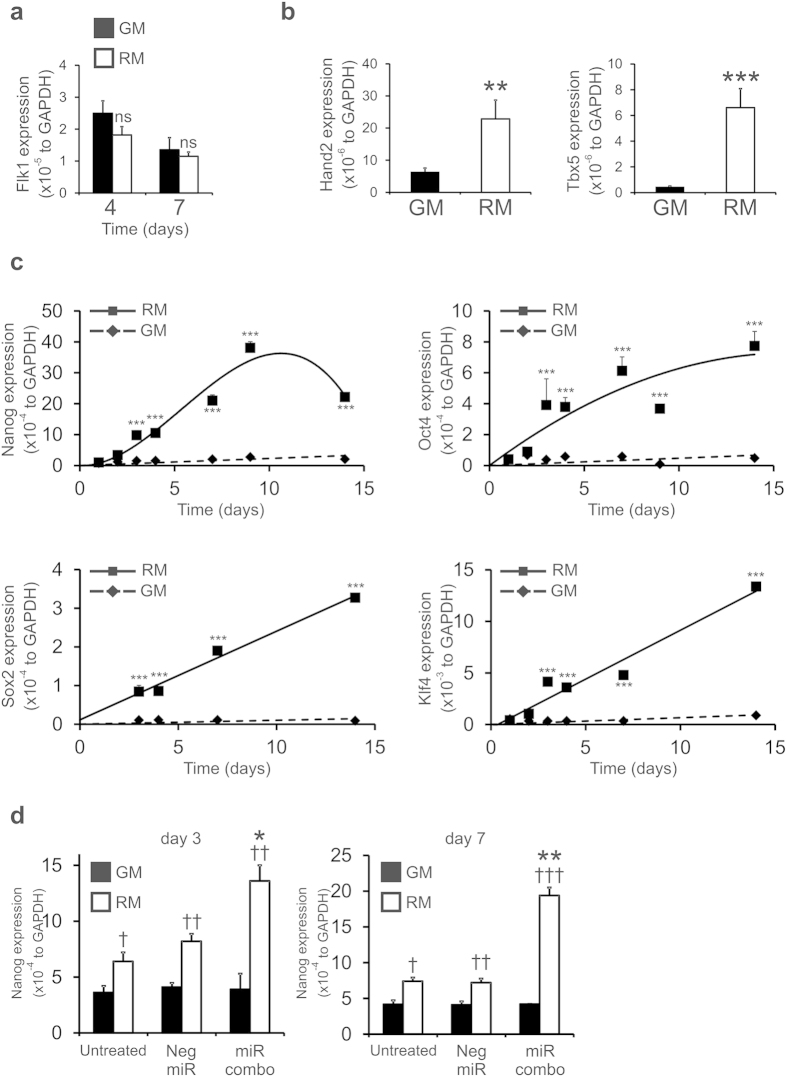
Reprogramming media induces pluripotency gene expression. Neonatal tail-tip fibroblasts were cultured in growth media (GM) or reprogramming media (RM) for the indicated times. qPCR was used to determine the expression of (**a**) cardiac progenitor, (**b**) cardiac commitment, and (**c**) pluripotency markers. N = 3–12. ***P < 0.001, **P < 0.01, *P < 0.05. Expression is represented as absolute transcripts normalized to the endogenous control GAPDH. (**d**) Neonatal cardiac fibroblasts were transfected with vehicle, negative control miR (Neg-miR) or miR combo. The day after transfection the cells were cultured in either growth media (GM) or reprogramming media (RM) for the indicated times. Nanog expression was determined by qPCR and is expressed relative to the endogenous control GAPDH. N = 3. *Comparisons made between miR combo and negative control miR **P < 0.01, *P < 0.05. ^†^Comparisons made between reprogramming media and growth media for each group ^†††^P < 0.001, ^††^P < 0.01, ^†^P < 0.05.

We then wished to determine the mechanism by which RM induced cardiac gene expression in neonatal fibroblasts. Flk-1 expression was not affected by RM suggesting that the neonatal tail-tip fibroblasts were not adopting a cardiac progenitor cell fate (Fig. 3a). RM induced the expression of Hand2 and Tbx5, two early markers of commitment to the cardiac lineage, by 3- and 12-fold respectively (Fig. 3b). More dramatic effects were observed with pluripotency markers. Nanog mRNA levels in RM treated neonatal tail-tip fibroblasts reached >100-fold above that of cells cultured in GM by day 7 (Fig. 3c). Similarly, RM induced Oct4 levels ~40-fold, Sox2 levels by ~30-fold, and Klf4 levels by ~25-fold (Fig. 3c). GM had no effect on the expression of these pluripotency genes at any time point (Fig. 3c). We verified the gene expression experiments by flow cytometry. There were significantly higher percentages of Oct4 and SSEA-1 positive cells in neonatal tail-tip fibroblasts cultured in RM when compared to the GM treated group (Supplementary Fig. 2a). Our data suggested that RM was inducing neonatal tail-tip fibroblasts to become iPS cells. To investigate this further, we cultured neonatal tail-tip fibroblasts in GM or RM for 14 days and re-plated the cells onto a MEF feeder layer. These co-cultures were cultured in standard iPSC culture media to measure colony formation. Colony formation was observed with neonatal tail-tip fibroblasts cultured in RM but not GM (Supplementary Fig. 2b). We observed 1–3 colonies per 10,000 cells plated. These colonies tested positive for various iPS markers such as alkaline phosphatase (Supplementary Fig. 2b), Nanog (Supplementary Fig. 2c) and Oct4 (Supplementary Fig. 2c) however expansion was very limited indicating that the colonies lacked the proliferative capacity of typical iPS cells (data not shown).

As described above Nanog expression increased dramatically in neonatal tail-tip fibroblasts cultured in RM. Similarly, RM increased Nanog expression in neonatal cardiac fibroblasts (Fig. 3d). MiR combo had no effect on Nanog expression when cells were cultured in GM. However, miR combo significantly augmented the effect of RM upon Nanog expression (Fig. 3d).

**Figure 4:**
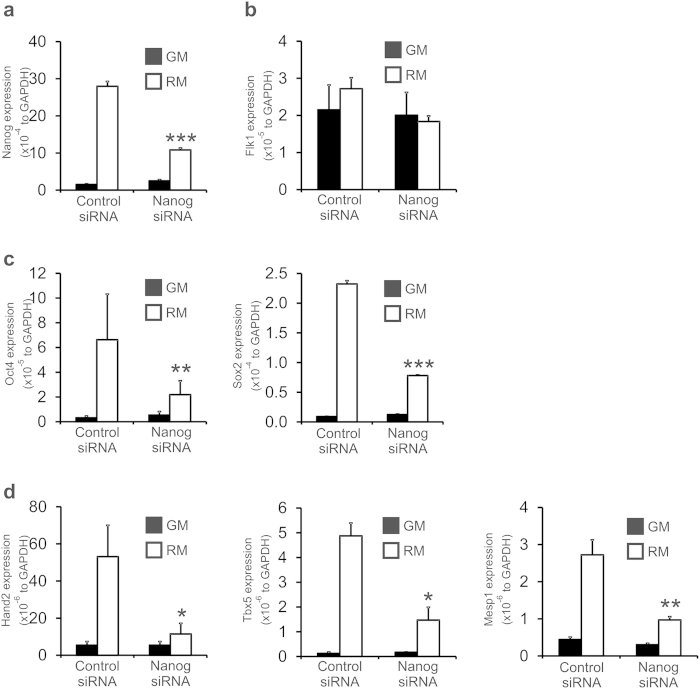
Nanog knockdown inhibits the effect of reprogramming media. (**a**) Nanog knockdown efficiency. Neonatal tail-tip fibroblasts were transfected with a negative control or Nanog siRNA. Nanog expression was determined by qPCR three days after transfection. (**b**–**d**) The effect of Nanog knockdown upon pluripotency, cardiac commitment and cardiac progenitor marker expression was determined by qPCR. N = 3. ***P < 0.001, **P < 0.01, *P < 0.05. Expression is represented as absolute transcripts normalized to the endogenous control GAPDH.

Taking these results above into consideration we wished to determine if Nanog was responsible for the effect of RM upon cardiac gene expression. Neonatal tail-tip fibroblasts were transfected with either a negative control or Nanog siRNA. Nanog knockdown was significant as determined by qPCR (Fig. 4a). Flk1 expression was not affected by Nanog siRNA (Fig. 4b). Knockdown of Nanog significantly inhibited the expression of the pluripotency markers Oct4 and Sox2 (Fig. 4c). Moreover, Nanog knockdown significantly inhibited cardiac gene expression in neonatal tail-tip fibroblasts cultured in RM for all the markers tested (Fig. 4d).

### Selenium is the active component of reprogramming media

**Figure 5:**
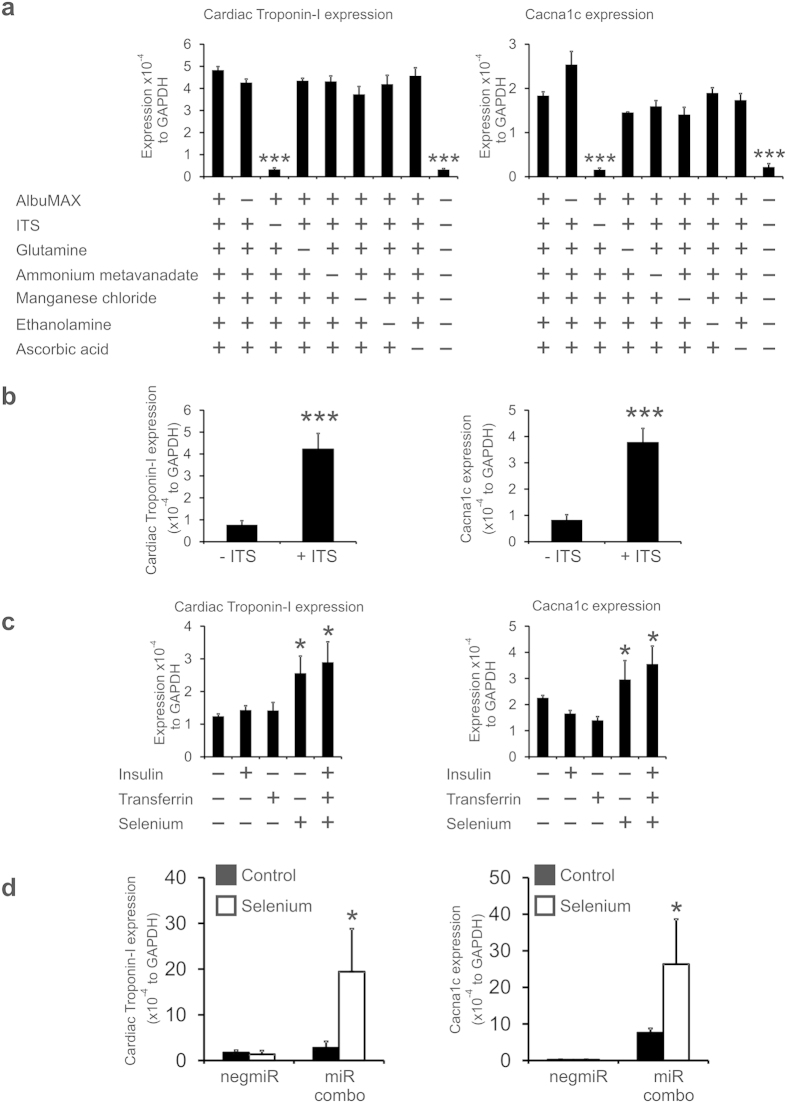
Insulin-transferrin-selenium is the active component of reprogramming media. (**a**) Neonatal tail-tip fibroblasts were cultured in the indicated medias for 14 days. Cardiac gene expression was determined by qPCR and is expressed relative to the endogenous control GAPDH. N = 3. ***P < 0.001. (**b**) Neonatal tail-tip fibroblasts were cultured in DMEM/F12 with and without ITS for 14 days. N = 3. ***P < 0.001. Expression is expressed relative to the endogenous control GAPDH. (**c**) Neonatal tail-tip fibroblasts were cultured in DMEM/F12 media with the indicated compounds for 14 days. Cardiac gene expression was determined by qPCR and is expressed relative to the endogenous control GAPDH. N = 3. *P < 0.05. (**d**) Neonatal cardiac fibroblasts were transfected with vehicle, negative control miR (Neg-miR) or miR combo. The day after transfection the cells were cultured in either growth media or growth media supplemented selenium for 14 days. Cardiac gene expression was determined by qPCR and is expressed relative to the endogenous control GAPDH. N = 3. *P < 0.05.

We then wished to determine the active component of RM. Our reprogramming media (RM) differs from standard DMEM:F12 by the inclusion of several specialized components: AlbuMAX, insulin-transferrin-selenium, ascorbic acid, glutathione, ammonium metavanadate, Manganese chloride, and ethanolamine. Neonatal tail-tip fibroblasts were cultured in DMEM:F12, RM, or RM lacking one of the aforementioned specialized components. Cardiac gene expression was assessed after 14 days by qPCR. Only the removal of insulin-transferrin-selenium completely inhibited the effect of RM upon cardiac gene expression (Fig. 5a). Importantly the addition of insulin-transferrin-selenium to DMEM:F12 induced Cardiac Troponin-I and Cacna1c expression (Fig. 5b). We then wished to determine which component of insulin-transferrin-selenium was promoting cardiac gene expression in fibroblasts. Neonatal tail-tip fibroblasts were cultured in DMEM:F12 or DMEM:F12 supplemented with insulin, transferrin, or selenium for 14 days and cardiac gene expression assessed by qPCR. Insulin and transferrin had no effect on cardiac gene expression (Fig. 5c). In contrast, selenium significantly increased cardiac gene expression (Fig. 5c). Moreover, the addition of selenium fully recapitulated the effects of insulin-transferrin-selenium (Fig. 5c). Finally, we wanted to determine if the addition of selenium enhanced the reprogramming efficiency of miR combo. To that end, neonatal cardiac fibroblasts were transfected with miR combo or the control negmiR. Standard growth medium was supplemented with selenium or equivalent volume of vehicle (water). After 14 days cardiac gene expression was determined by qPCR. In standard growth media miR combo significantly induced the expression of the cardiomyocyte markers cardiac troponin-I and Cacna1c (Fig. 5d). The addition of selenium to the growth media enhanced the effect of miR combo; expression of cardiac troponin-I and Cacna1c were enhanced by 10- and 4-fold respectively (Fig. 5d).

## Discussion

We have identified a chemical defined media, RM, which augments the ability of miR combo to directly reprogram fibroblasts into cells with a cardiac phenotype.

Combinations of transcription factors^7^,^8^,^10^ or microRNAs (miR combo)^3^,^4^,^5^ have successfully reprogrammed fibroblasts directly into cardiomyocytes. Both of these direct reprogramming methodologies give rise to improvements in cardiac function when delivered into the mouse heart following myocardial infarction^3^,^4^,^7^,^8^. Despite these encouraging results direct reprogramming suffers from low efficiency which limits future clinical applications. Thus, there is a need to identify strategies that improve the efficiency of direct reprogramming. Our study shows that the addition of selenium improves the efficiency of direct reprogramming. Selenium is known to be important for cell proliferation^11^ and is essential for sustained ESC culture expansion^12^. The necessity of selenium for the maintenance of ESC cultures may partly explain the iPS-like colonies that form in response to RM. It has been known for a number of decades that selenium plays a beneficial role in human health^13^. This is attributed to the presence of selenium in a number of proteins, named selenoproteins, in the form of the amino acid selenocysteine^13^. Such selenoproteins are known to be important for the formation of muscle and normal heart function. MyoD, the master regulator of skeletal muscle differentiation, activates the Selenoprotein W gene^14^. Selenoprotein W subsequently promotes skeletal muscle differentiation by inhibiting TAZ binding to 14-3-3 protein^15^. Several selenoproteins have been shown to have extensive roles in the heart^16^. For example, ablation of GPx1 leads to increased infarct size following injury^17^ and cardiac tissue restricted ablation TrxR2 results in fatal dilated cardiomyopathy^18^. It is possible that such selenoproteins may be involved in the transdifferentiation of fibroblasts into cardiac-like cells by RM and it is tempting to speculate as to what effect selenium would have upon direct reprogramming efficiency *in vivo*. Beyond selenium, other small molecules have been used to directly reprogram fibroblasts into cardiomyocytes. The TGFβ pharmacological inhibitor SB431542 has been shown to augment transcription factor mediated reprogramming^19^. Furthermore, SB431542 in combination with the pharmacological inhibitors CHIR99021, forskolin and parnate reprogrammed tail-tip fibroblasts into cardiomyocytes in the presence of the transcription factor Oct4^20^.

Finally the relative ease with which fibroblasts, cardiac and tail-tip, can be forced to express cardiac genes suggests that these cells possess an inherent plasticity. Other researchers have come to similar conclusions. Using fate-mapping techniques cardiac fibroblasts were shown to adopt an endothelial cell phenotype after cardiac injury suggesting that adult fibroblasts possess an intrinsic plasticity^21^.

## Methods

### Chemically defined reprogramming media

Mouse (C57BL/6) neonatal cardiac fibroblasts or tail tip fibroblasts were isolated from 2 day old mouse neonates according to the method outlined in Jayawardena *et al.*^5^. Following isolation fibroblasts were cultured in growth media containing DMEM (ATCC, Catalogue number 30–2002) supplemented with 15%v/v FBS (Thermo Scientific Hyclone Fetal bovine serum, Catalogue number SH30071.03, Lot number AXK49952) and 1%v/v penicillin/streptomycin (Gibco, Catalogue number 15140-122, 100 units Penicillin, 100 ug/ml Streptomycin). Fibroblasts were passaged once the cells had reached 70–80% confluence by incubating the cells for five minutes with 0.05% w/v trypsin (Gibco, Catalogue number 25300-054). Freshly isolated fibroblasts were labelled as Passage 0. Experiments were conducted with cells at passage 1 or passage 2. For all experiments, cells were seeded at 5000 cells/cm^2^ in growth media. Twenty-four hours later growth media was replaced with chemically defined reprogramming media. This chemically defined reprogramming media was comprised of a basal media, DMEM/F12 (Gibco, Catalogue number 21331-020), to which the following components were added: AlbuMAXII (Gibco, Catalogue number 11021-029, 400 mg/L), BSA (0.2%w/v), Ascorbic Acid (250 μM), Insulin (Gibco, Catalogue number 12585-014, 10 mg/L), Transferrin (Gibco, Catalogue number 11107-018, 5.5 mg/L), Sodium Selenite (Sigma Aldrich, Catalogue number S5261, 6.7 ug/L), Glutathione (Sigma Aldrich, Catalogue number G6013, 1 mg/L), Ammonium metavanadate (Sigma Aldrich, Catalogue number 205559, 0.3 ug/L), Manganese chloride (Sigma Aldrich, Catalogue number 48795, 0.05 ug/L), Ethanolamine (Sigma Aldrich, Catalogue number E0135, 1.9 mg/L), L-Glutamine (1×, Gibco, Catalogue number 35050-061, 2 mM Glutamine), Penicillin-Streptomycin (1×, Gibco, Catalogue number 15140-122, 100 units Penicillin, 100 ug/ml Streptomycin). Every two days media was removed and replaced with fresh media.

### MicroRNA transfection

Fibroblasts were seeded into 24 well plates at 9,000 cells per well. After 24 hours, the cells were transfected with transfection reagent alone (Dharmafect-I, ThermoScientific), with transfection reagent plus non-targeting microRNAs (negmiR), or with transfection reagent plus our previously reported combination of cardiac reprogramming microRNAs^3^ (miR combo, miR-1, miR-133, miR-208, miR-499). The transfection method has been previously reported by Jayawardena *et al.*^5^. One day after transfection the media was removed and replaced with either growth media or reprogramming media. The media was removed and replaced with fresh media every two days.

### iPS cell culture

Cells were cultured in CTS^TM^ KnockOut^TM^ DMEM (Gibco, Catalogue number A12861-01) supplemented with 15%v/v fetal bovine serum (Thermo Scientific Hyclone Fetal bovine serum, Catalogue number SH30071.03, Lot number AXK49952), 1× GlutaMAX^TM^ (Gibco, Catalogue number 35050-061), 1× MEM non-essential amino acids (Gibco, Catalogue number 11140050), 1% v/v penicillin/streptomycin (Gibco, Catalogue number 15140-122), 0.0007% v/v β-mercaptoethanol (Gibco, Catalogue number 21985023), and 0.1 U/mL LIF (Leukemia Inhibitory Factor, Stem Cell Technologies, Catalogue number 02642).

### qPCR

Total RNA was extracted using Quick-RNA MiniPrep Kit according to the manufacturer’s instructions (Zymo Research). Total RNA (50–100 ng) was converted to cDNA using a high capacity cDNA reverse transcription kit (Applied Biosystems). cDNA was used in a standard qPCR reaction involving FAM conjugated gene specific primers and TaqMan Gene Expression Master Mix (Applied Biosystems). The following primers were used for qPCR: Gapdh (Mm99999915_m1), Nanog (Mm02384862_g1), Oct4/Pou5f1 (Mm03053917_g1), Rexo1 (Mm00617735_m1), Sox2 (Mm03053810_s1), Klf4 (Mm00516104_m1), Tnni3 (Mm00437164_m1), Actn2 (Mm00473657_m1), Cacna1c (Mm00437917_m1), Scn5a (Mm00451971_m1), Mesp1 (Mm00801883_g1), Nkx2-5 (Mm00657783_m1), Tbx5 (Mm00803518_m1), Hand2 (Mm00439247_m1), Gata6 (Mm00802636_m1), Sox17 (Mm00488363_m1), Pax2 (Mm01217939_m1), Pax6 (Mm00443081_m1), Pecam-1 (Mm01242576_m1) and Kdr/Flk-1 (Mm01222421_m1). Gene expression data is expressed as relative to the endogenous control GAPDH (2^−ΔCT^ where ΔCT = C_T_ gene of interest–C_T_ GAPDH)^22^.

### Flow cytometry

Cells were seeded in 6-well plate at 40,000 cells/well and fixed, when appropriate, with 4%v/v paraformaldehyde for 15 minutes at 4 °C. Cells were washed with FACS buffer (1 × PBS, 2 mM EDTA, 5%w/v BSA, 0.2%w/v saponin) at 800 g for 5 minutes at 4 °C. Following washing cells were incubated in FACS buffer for 1 hour at 4 °C with the following antibodies (0.4 μg/10^6^ cells): αMHC (Abcam, ab15), αSarcomeric Actinin (Abcam, Ab68167), cardiac troponin-T (Abcam, FITC conjugate, ab105439), SSEA-1 (StemGent 09-0005), Oct4 (Abcam, ab18976). Following antibody incubation cells were washed once in FACS buffer. Where necessary, cells were incubated with a secondary anti-rabbit PE or anti-mouse APC conjugate in FACS buffer for 1 hour at 4 °C (1:1000 dilution). Cells were washed and re-suspended in FACS buffer. Cells were analyzed by FACS on a FACSCantoII (BD Biosciences). FlowJo version 10 was used to compensate and analyze the data.

### Immunofluorescence

Cells were fixed with 2%v/v paraformaldehyde (EMS) as described previously^23^. Fixed cells were blocked in antibody buffer (1%w/v BSA, 0.3%v/v Triton X-100, in PBS) for 1 hr at room temperature and then incubated with primary antibodies overnight at 4 °C in antibody buffer. αMHC (Abcam, ab15, 1:200), cTnI (Abcam, ab47703, 1:400), Oct4 (Abcam, ab18976, 1:300), and Nanog (Abcam, ab14959, 1:100) were used at indicated concentrations. Alexa-Fluor conjugated secondary antibodies (Invitrogen) were used at 1:1000 dilution in antibody buffer for 1 hr at room temperature. Nuclei were stained by DAPI at 1 μg/ml for 15 minutes at room temperature in PBS.

### Nanog siRNA knockdown

A Nanog siRNA pool (four siRNAs targeting Nanog) and a negative control were purchased from Dharmacon. siRNAs were made to 20 μM in nuclease free water, aliquoted, and stored −80 °C until use. Fibroblasts were seeded into 24 well plates at 9,000 cells per well one day prior to transfection. On the day of transfection siRNAs were diluted to 5 μM in nuclease free water. For each well 5 μl of the working siRNA solution was diluted with 95 μl Optimem-Serum Free media (Gibco, Catalogue number 31985-070). In a separate tube 5 μl of dharmafect-I (Dharmacon, Catalogue number T-2001-02) was diluted with 95 μl Optimem-Serum Free. After a 5 minute incubation the two solutions were combined. After 20 minutes growth media lacking antibiotics was added (800 μl) and the transfection complexes added to the cells.

### Statistics

Statistical analysis was performed with GraphPad or R. Experiments containing 2 conditions a t test was performed. ANOVA was used for experiments with ≥3 conditions followed by Bonferroni post hoc tests for comparisons between individual groups.

## Additional Information

**How to cite this article**: Wang, X. *et al.* Selenium Augments microRNA Directed Reprogramming of Fibroblasts to Cardiomyocytes via Nanog. *Sci. Rep.* **6**, 23017; doi: 10.1038/srep23017 (2016).

## Supplementary Material

Supplementary Information
