# Supplementary material for: Selenium Augments microRNA Directed Reprogramming of Fibroblasts to Cardiomyocytes via Nanog
Source: Sci Rep. 2016 Mar 15;6:23017. doi: 10.1038/srep23017 (PMC4792153; doi:10.1038/srep23017)
Supplement: Supplementary Information [file srep23017-s1.pdf]

SUPPLEMENTARY

Selenium Augments microRNA Directed Reprogramming of Fibroblasts to Cardiomyocytes via Nanog

Xiaowen Wang BA[1], Conrad P Hodgkinson PhD[1], Kefeng Lu MD PhD[1,2], Alan J Payne BSc[1], Richard E Pratt PhD[1], Victor J Dzau MD[1]

Supplementary Figures

Supplementary Figure 1. Reprogramming media does not affect endodermal, ectodermal and vascular markers. Neonatal cardiac fibroblasts were transfected with vehicle, negative control miR (Neg-miR) or miR combo. The day after transfection the cells were cultured in either growth media (GM) or reprogramming media (RM) for the indicated times. Expression of endodermal, ectodermal and vascular markers was determined by qPCR and is expressed relative to the endogenous control GAPDH. N=3. *Comparisons made between miR combo and negative control miR ***P<0.001, **P<0.01, *P<0.05. †Comparisons made between reprogramming media and growth media for each group †††P<0.001, ††P<0.01, †P<0.05.

Supplementary Figure 2. Reprogramming media promotes the formation of iPS-like state.
(**a**) Neonatal tail-tip fibroblasts were cultured in growth media (GM) or reprogramming media (RM) for 7 or 14 days. Cells were stained Oct4 or SSEA-1 antibodies and analyzed by flow cytometry. N=3. ***P<0.001, **P<0.01, *P<0.05.
(**b**) Neonatal tail-tip fibroblasts were cultured in growth media (GM) or reprogramming media (RM) for 14 days and then plated on a MEF layer. Colonies were counted and assayed for alkaline phosphatase staining. N=3. Scale bar 100 microns. Representative images are shown.

(**c**) Neonatal tail-tip fibroblasts were cultured in growth media (GM) or reprogramming media (RM) for 14 days. The cells were fixed and immunostained for Nanog (left panels) and Oct4 (right panels). N=3. Scale bar 100microns. Representative images are shown.

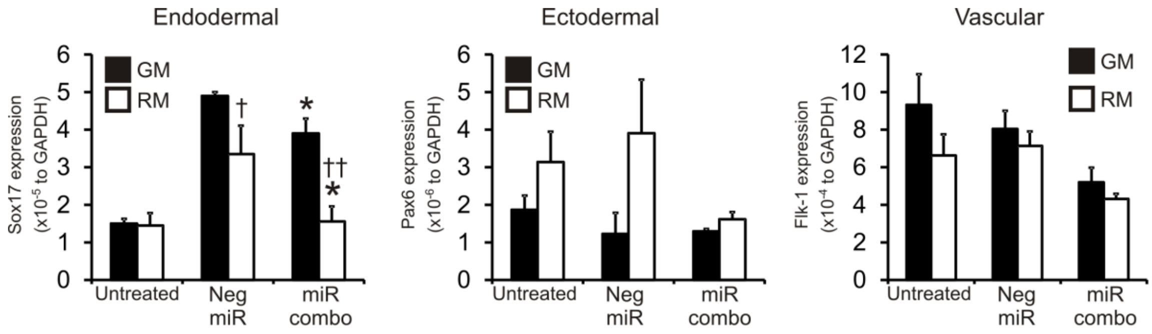

Supplementary Figure 1

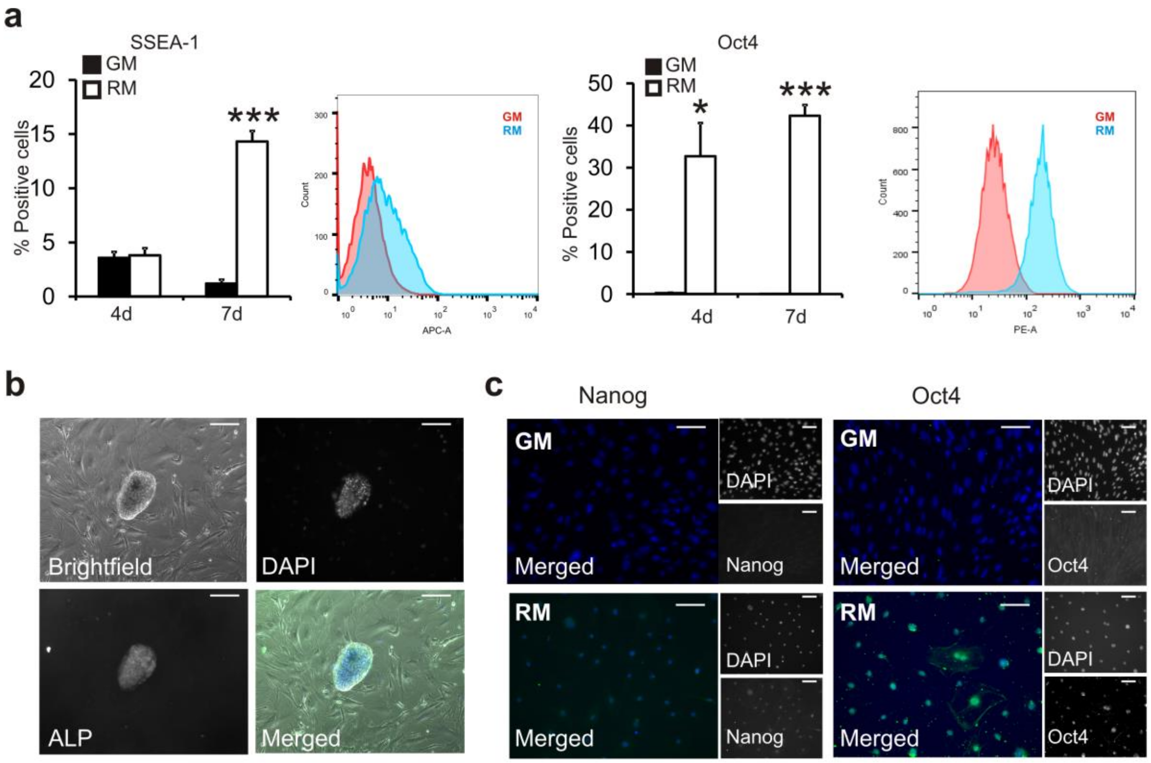

Supplementary Figure 2
